# Supplementary material for: Intrinsic ecological dynamics drive biodiversity turnover in model metacommunities
Source: Nat Commun. 2021 Jun 15;12:3627. doi: 10.1038/s41467-021-23769-7 (PMC8206366; doi:10.1038/s41467-021-23769-7)
Supplement: Supplementary file 1 — Supplementary information file [file 41467_2021_23769_MOESM1_ESM.pdf]

SUPPLEMENTARY INFORMATION FILE

Distribution of interaction coefficients

In simulations described in the main text, we sampled interspecific interaction coefficients from a discrete random distribution chosen for its simplicity and relative computational efficiency. Previous work[1–3] has shown that macroscopic structures, including ecological limits due to the onset of structural instability, are determined by the mean and variance of the off-diagonal elements of the interaction matrix (assuming the diagonal is normalised to one), and are largely independent of finer details of the statistical distribution of entries. The discrete distribution we chose for these entries is perhaps the simplest choice for which many entries are zero (implying a sparse interaction network as typically observed in nature), that excludes non-negative terms, and is asymmetric. We expect that alternative models which preserve these basic properties, or even slightly violate them, will produce the same outcomes.

To demonstrate that this is indeed true for the model described above, we assembled metacommunities of $N = 32$ sites, spatial parameters as in main text Fig. 5, but with the entries A_{ij} sampled as products of two random variables X and Y. We sampled X from either a Beta distribution or a normal distribution, and Y as a discrete random variable with $P(Y = 1) = 0.4$ (i.e., a connectance of 0.4) and 0 otherwise. The distributions X were parameterised such that the product distribution XY preserved the expectation and variance of the discrete model used in main text Fig. 5, while keeping the matrices relatively sparse. This meant a coefficient of variation of 0.58 of the non-zero interspecific interaction terms, i.e. a fairly broad spread of values. Note that there exists no combination of parameters which preserve the mean, variance *and* connectance of the discrete model, and for which the coefficient of variance of non-zero terms is greater than zero.

As shown in Figs. 2a and b, autonomous turnover emerges in these modified metacommunity models once local communities become ecologically saturated, irrespective of the distribution from which interaction coefficients were sampled. This demonstrates that autonomous turnover arises irrespective of our choice for the distribution of interaction coefficients (Fig. 2c), and that the triggering mechanism is the same.

The simulation data used to generate Fig. 2 is publicly available[4], while example scripts for their generation are included in the software of the LVMCM.

The variance and, to some extent, the mean of the distribution of off-diagonal elements of

$\mathbf{A}$ determine the local species richness at which community saturation occurs[1]. To address the question whether our choice of these parameters was realistic, we note that, in real systems, the magnitude of the effective interspecific interaction coefficients A_{ij} depends, somewhat arbitrarily, on the spatial extent of the "local patches". As we have shown in previous work[5], effective interaction matrices for systems obtained by merging several small patches have smaller off-diagonal entries than the interaction matrices controlling the small patches themselves, and as a result these larger, merged communities can harbour a larger number of species. As such, the distribution of A_{ij} implicitly reflects the area of the local community, which we arbitrarily fixed for all sites in the model described above.

Spatial parameterization

Other than patch number N, the parameters that most impact the spatio-temporal structure of model metacommunities are the environmental correlation length ϕ, the variability of the environment σ^2, and the dispersal length ℓ. In order to understand the role of these parameters for autonomous turnover, we fixed $N = 64$ and assembled metacommunity models with $\sigma^2, \ell \in \{1 \times 10^{-2},\ 5 \times 10^{-2},\ 1 \times 10^{-1},\ 5 \times 10^{-1},\ 1\}$, and $\phi \in \{1,\ 5,\ 10,\ 50,\ 100\}$ in all combinations and computed the resulting temporal beta diversity as the mean spatially averaged temporal BC dissimilarity observed in 10 replicates of each parameterization. Rates of autonomous turnover varied in a complex but systematic way under variation in the spatial parameterization of the model, with turnover being weakly correlated with the dispersal length and maximized for intermediate habitat heterogeneity and autocorrelation (Fig. 4). Weak abiotic heterogeneity seeds the non-uniform spatial structure of the metacommunity and therefore promotes turnover. For large enough spatial networks, dispersal limitation and competitive repulsion alone are sufficient to drive autonomous dynamics in perfectly uniform landscapes. The scan of the parameter space allowed selection a parameterization with strong autonomous turnover: $\phi = 10$, $\sigma^2 = 0.01$, $\ell = 0.5$ (peak in Fig. 4a). Using this combination of parameters we then assembled metacommunity models of $N = 8,\ 16,\ 32,\ 48,\ 64, 80,\ 96,\ 128,\ 160,\ 192,\ 224,\ 256$ patches.

To some extent, the complex roles of parameters ϕ, σ^2, and ℓ, shown in Fig. 4, can be distilled into the effect on a single parameter: the average spatial community dissimilarity at the local neighbourhood scale. This is due to the fact that the impact of each of the parameters, which control the between-patch differences in environment and the strength of mass effects, is reflected

in the degree of spatial beta diversity within the metacommunity. To demonstrate this we used the multiple-site dissimilarity metric derived in Ref.[6], which generates an unbiased total beta diversity metric for systems of three or more sites/time points. Since both local neighbourhood and (correspondingly) temporal turnover vary within a given metacommunity, we show the beta diversity metrics averaged over all patches.

Temporal turnover responded unimodally to local neighbourhood dissimilarity (Fig. 11) over the parameter range of Fig. 4, suggesting that spatial parameterisations that maximise β_s, either through exaggerating abiotic differences between adjacent local communities or dampening mass effects, can elevate neighbourhood diversity while simultaneously suppressing the pool of species that can actually invade.

This result makes plausible why empirical studies have detected a range of statistical associations between spatial and temporal turnover in natural ecosystems. Positive, negative, unimodal, and non-significant relationships have been reported between temporal turnover and species richness or spatial turnover[7–12]. The unimodal response shown in Fig. 11 may help to resolve these apparent contradictions: it is not species richness or spatial dissimilarity *per se* that best predict temporal turnover, but the size of the pool of species capable of passing through biotic and abiotic filters to invade a local community.

Phase space of a generalised Lotka-Volterra community

Analytic theory[2] predicts a sharp transition between what has been called the Unique Fixed Point (UFP) and Multiple Attractor (MA) phases. In Fig. 7 we reproduce the phase portrait for such a system and note that our explicitly modelled metacommunities reveal a gradual transition in the MA phase space from oscillatory, to Clementsian and into Gleasonian turnover regimes. Assuming large S, the sharp transition between UFP and MA phases has been shown[2] to occur at species richness

$$S = \frac{2}{(1+\gamma)^2 \operatorname{var}(A_{ij})}, \tag{1}$$

where $\gamma = \operatorname{corr}(A_{ij}, A_{ji})$ denotes the degree of correlation in the effects two species have on each other, measuring the symmetry of interspecific interaction strengths, and $\operatorname{var}(A_{ij})$ is the variance in the distribution. In our model we use a random interaction matrix for which $\gamma = 0$. We sample interaction coefficients from a discrete distribution with $\operatorname{var}(A_{ij}) = (0.25)^2$ giving

a predicted transition into the MA phase space at $S = 32$ species. Thus, while the prediction is approximate for small S communities with non-uniform intrinsic growth rates, a numerically observed threshold of around 35 species in the isolated LV model (main text Fig. 4c inset) is consistent with these analytic predictions.

Isolated LV communities

To explore the emergence of heteroclinic networks in LV models, we studied an isolated LV model with and without coupling to an implicitly modelled neighbourhood species pool. The dynamics of the model follow

$$\frac{d\mathbf{b}}{dt} = \mathbf{b} \circ (\mathbf{r} - \mathbf{A}\mathbf{b}) + \boldsymbol{\epsilon}, \tag{2}$$

where $\mathbf{b}$ is a population biomass vector of length S, $\mathbf{r}$ is a vector of independent random normal variables with mean 1 and variance $\sigma^2 = 0.01$ representing maximum intrinsic growth rates, $\mathbf{A}$ is a competitive overlap matrix and the vector $\boldsymbol{\epsilon}$ represents the slow immigration of biomass corresponding to a weak propagule pressure. The elements ϵ_i are analogous the to explicitly modelled immigration terms $B_{ix}D_{xy}$ of the full metacommunity model.

As in the metacommunity model, interspecific competition coefficients A_{ij} were set to 0.5 with a probability of 0.5 for $i \neq j$ and otherwise to zero, while $A_{ii} = 1$, for all i. We enforced $b_i > 0$ for all i by simulating dynamics in terms of logarithmic biomass variables. In simulating this model, we did not follow the common practice of removing species whose biomass drops below some threshold. Instead all species were retained. We consider two situations: with and without the inclusion of a weak propagule pressure $\boldsymbol{\epsilon}$.

Heteroclinic networks in the case without propagule pressure: We first demonstrate in simulations that, indeed, as predicted under certain constraints[13], stable heteroclinic networks exist in the MA phase of model equation (1) for $\boldsymbol{\epsilon} = 0$. For this we choose $S = 300$, which, with other parameters set as described above, brings us deeply into the MA phase of the model. Simulations were initialised by setting all $B_i = 10^{-3}$ $(1 \leq i \leq S)$ at $t = 0$. The system was simulated until $t = 2.1 \cdot 10^7$ and system states recorded at times $t = 2.1 \cdot 10^{j/1000}$ $(0 \leq j \leq 7000)$. As illustrated in Fig. 8, while dynamics tend to become slower for larger t, no stable equilibrium or other simple attractor appears to be ever reached—as expected for a system approaching a heteroclinic network. Instead, as expected when a heteroclinic network exists, the system bounces around between un-

stable equilibria, apparently in a random fashion. Unexpected to us, however, the system appears to visit not only unstable equilibria in its transient, but occasionally also unstable periodic orbits ($t \approx 1.3 \cdot 10^4$ in Fig. 8) and perhaps more complex invariant sets ($t \approx 1.2 \cdot 10^6$ in Fig. 8).

One might wonder whether there is any tendency for dynamics to eventually come to a halt. To study this question, we calculated the number of changes in community composition (species colonisations and extinctions) between all pairs of subsequently recorded system states, where we considered a species i as "present" if $B_i > 10^{-4}$, and from this the momentary rate of change in composition on the $\ln(t)$ scale by dividing by $\ln(10^{1/1000})$. In Fig. 9 we show the time series of the centred moving average over this number for 100 subsequent pairs or recordings, and averages for non-overlapping adjacent blocks for 300 pairs. Spikes where the rate of change is particularly high correspond to brief phases of regular or irregular oscillation. We performed a median regression of the block-wise averages by a power law of the form: (rate) $\sim t^{\nu}$. Median regression was used to de-emphasise the spikes. For the simulation shown in Fig. 8 we found that ν did not differ significantly from zero, implying a decline of the turnover rate on the natural time axis as t^{-1}. When we repeated this analysis for 15 independent simulations (two of which failed due to numerical issues), we observed a tendency for ν to be slightly positive ($\nu = 0.054 \pm 0.020$, t-test $t = 2.67$, $p = 0.020$), perhaps because the effect of oscillatory phases on the mean turnover rate on the $\ln(t)$-scale increases with increasing t. Overall, however, the decline of turnover rate approximately as t^{-1} was confirmed, providing evidence for the existence of an attracting heteroclinic network that the LV system equation (2) with $\boldsymbol{\epsilon} = 0$ slowly approaches.

Use of logarithmic biomass variables was essential for these simulations. We found that median species biomass at the end of each run was typically around $10^{-3,500,000}$, much smaller than the smallest number representable by double precision floating point arithmetic, which is around $2 \cdot 10^{-308}$. Needless to say, these small numbers mean that the simulations with $\epsilon = 0$ are, while instructive, ecologically unrealistic.

Heteroclinic networks in the case with propagule pressure: The case $\boldsymbol{\epsilon} > 0$, where dynamics move alongside the underlying heteroclinic network without ever fully approaching it, is discussed in the Main Text as it provides a useful intermediate between the explicit metacommunity model and the more tractable isolated community. In Fig. 10 we show that the transition from oscillatory to Clementsian and finally Gleasonian turnover regimes can also be observed in these isolated LV models ($\epsilon_i = \epsilon = 10^{-15}$ for all i, other parameters as above).

Local structural instability drives autonomous turnover

Species richness in competitive LV communities is intrinsically limited by the onset of ecological structural instability. Here we show analytically that for isolated communities the boundary between the UFP and MA phases[2] is identical to the structurally unstable limit[1].

The transition between UFP and MA phase for competitive LV models occurs[2] when

$$\Phi = (u - \gamma v)^2 , \tag{3}$$

where $\Phi := S^*/S$ is the proportion of species persisting, i.e. the ratio between the number S^* of species that persist and the pool size S, and again $\gamma = \mathrm{cor}\,(A_{ij}, A_{ji})$. The quantities u and v in equation (3) are given by

$$u = \frac{1 - \mathsf{E}[A_{ij}]}{S^{1/2}\,\mathrm{std}\,(A_{ij})}, \tag{4}$$

with $\mathsf{E}[A_{ij}]$ and $\mathrm{std}\,(A_{ij})$ denoting mean and standard deviation of the distribution of off-diagonal entries of $\mathbf{A}$, respectively, and

$$v = \frac{\Phi}{u - \gamma v}. \tag{5}$$

For $\gamma \neq 0$, equation (5) does not have a unique solution for v. The equivalent quadratic equation $\gamma v^2 - uv + \Phi = 0$ has two solutions, one of which diverges as $\gamma \to 0$; this we discard. The other solution is

$$v = \frac{u - \sqrt{u^2 - 4\gamma\Phi}}{2\gamma}, \tag{6}$$

which becomes $v = \Phi/u$ for $\gamma \to 0$, consistent with equation (5). Substitution of equation (6) into equation (3) gives

$$\Phi = \left(\frac{u - \sqrt{u^2 - 4\gamma\Phi}}{2}\right)^2 , \tag{7}$$

which can be shown in a standard calculation to be equivalent to

$$\Phi = \frac{u^2}{(1+\gamma)^2} \tag{8}$$

for $u > 0$ and $-1 < \gamma < 1$. Finally, substituting equation (4) into equation (8) gives

$$S^* = \frac{\left(1 - \mathsf{E}[A_{ij}]\right)^2}{(1+\gamma)^2 \operatorname{var}(A_{ij})}, \tag{9}$$

which is exactly the theoretical limit of structural instability in isolated LV communities [equation (18.3) of Ref.[24]], thus demonstrating that UFP-MA phase boundary and the onset of structural instability perfectly coincide.

Temporal patterns in community structure

Fluctuations in local population biomasses as communities move between unstable equilibria in heteroclinic networks can span multiple orders of magnitude (red trajectories in Fig. 12a) and lead to significant temporal turnover in community composition (Fig. 12b). In contrast, the high-level properties of the assemblages remain largely unchanged. This is evident in the dampening of biomass fluctuations at metapopulation and metacommunity scales via a spatial portfolio effect[14–16] (blue and black trajectories in Fig. 12a), but also in the robustness of species biomass distribution (Fig. 12c) and range size distribution (Fig. 12d, range sizes computed as in Ref.[5]). In this case the mean relative biomass and range size are plotted irrespective of species identity (black lines) along with the mean ± one standard deviation (grey lines), for direct comparison with Ref.[17]. The relatively small standard deviations demonstrate a temporally robust distribution of metapopulation biomasses and spatial ranges, despite large fluctuations at the local scale.

STAR in large metacommunity models

We characterised the within assemblage STAR using a moving spatio-temporal window as described in the main text and comparing the resulting SAR and STR exponents. In Fig. 13 we show the nested SAR and STR for a single metacommunity of $N = 256$. The number of species detected for large spatial or temporal windows necessarily saturates in closed systems. We therefore defined the exponents of the STAR, displayed in Fig. 6 of the main text, as the maximum slope of the SAR/STR on double logarithmic axes.

SUPPLEMENTARY FIGURES

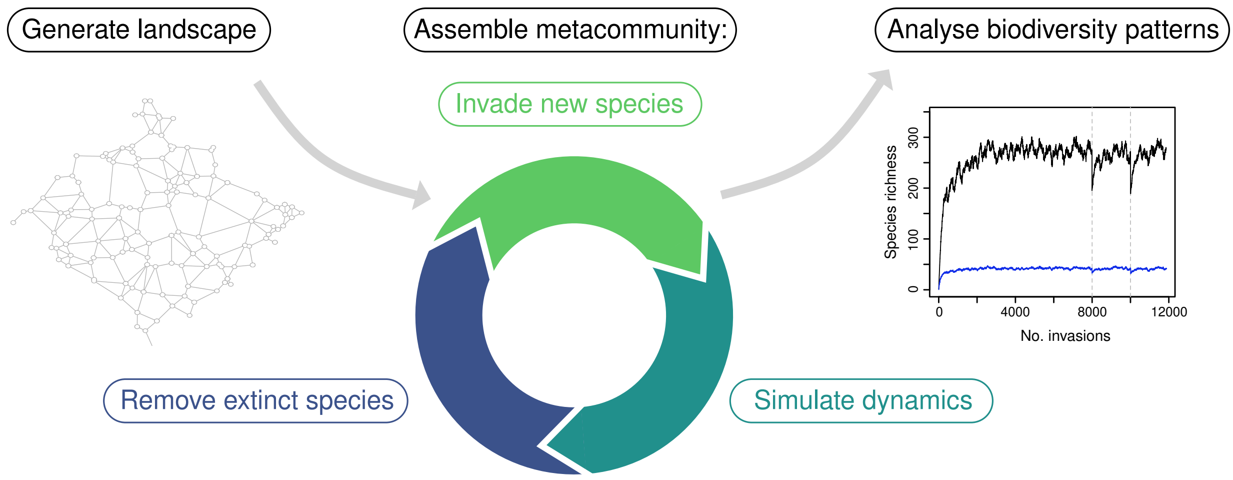

Supplementary Figure 1. **The metacommunity assembly algorithm.** First, a random planar graph is generated with spatial coordinates sampled at random and patches connected via the Gabriel[?] algorithm. Communities are then assembled iteratively: species are generated with intrinsic growth rates and interaction coefficients sampled from random distributions, introduced into the metacommunity at low abundance, metacommunity population-dynamics are simulated, and regionally extinct species are removed from the model before the next iteration. Eventually the metacommunity reaches both its local and regional diversity limits, the situation studied in the main text. In the inset a single metacommunity assembly process is shown; the black line represents regional species richness, the blue line average local species richness. Both are intrinsically regulated, as demonstrated by the effect of random removals of species (dashed lines) and subsequent re-assembly: local richness is barely affected and regional richness returns to the approximate same level. Inset adapted from Ref.[5]. See text for detailed description.

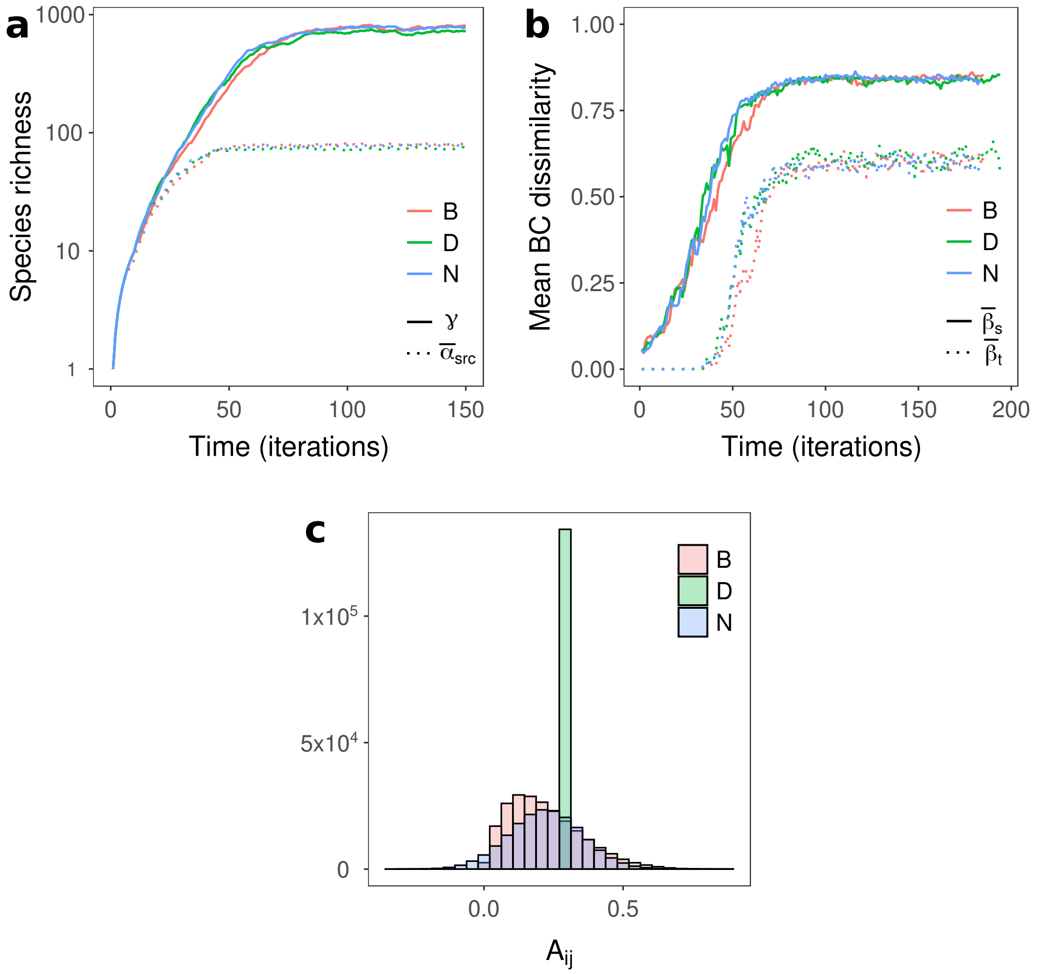

Supplementary Figure 2. **Autonomous turnover in different random matrix metacommunities.** **a**: Metacommunity assemblies with A_{ij} sampled from a Beta distribution with connectance 0.4 (B); a discrete distribution with $P(A_{ij} = 0.3) = 0.3$ as in main text Fig. 5 (D); and a normal distribution with connectance 0.4 (N). In all cases, the mean and variance of the fundamental distributions were identical. Shown are the regional diversity γ and the mean local richness of source populations $\bar{\alpha}_{src}$ since this is the community component directly subject to ecological limits. Here, in each iteration of the assembly model (regional invasion event), $0.1S + 1$ species were introduced and a total 10^4 invasions were simulated. **b**: As shown in main text Fig. 5, autonomous turnover sets in once local communities become saturated with respect to species richness. **c**: The realised distributions in *non-zero* interaction coefficients $\mathbf{A}_{ij}$ after metacommunity assembly. For visual clarity $\mathbf{A}_{ij} = 0$, representing 71.4%, 61.1% and 61.4% of interspecific interaction coefficients for the D, B and N distributions respectively, have been removed.

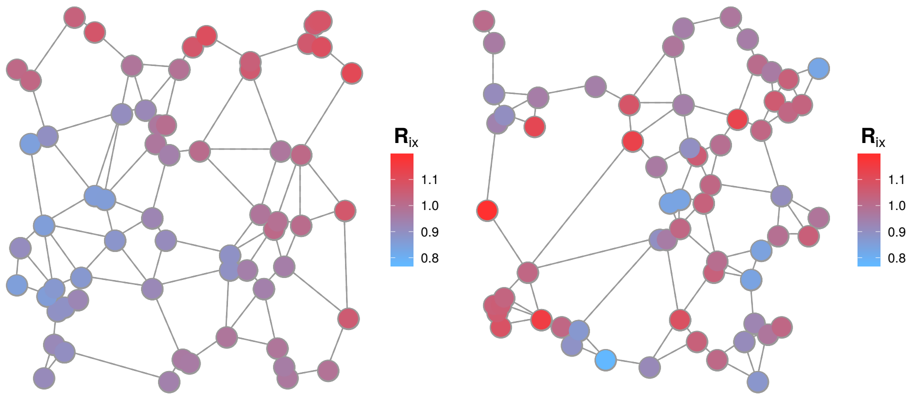

Supplementary Figure 3. **Spatially autocorrelated growth rate distributions.** Instrinsic growth rates are sampled from spatially autocorrelated random fields of autocorrelation length ϕ and variance σ^2. Two example distributions are shown, both for $N = 64$, $\sigma^2 = 0.01$, with $\phi = 10$ (left) and $\phi = 1$ (right). See Materials and Methods for details.

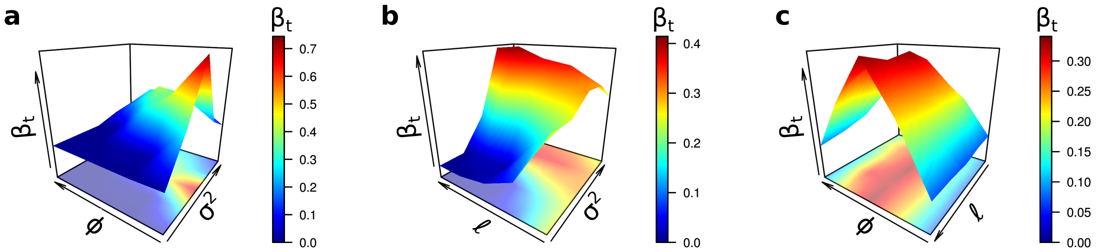

Supplementary Figure 4. **Temporal turnover throughout the spatial parameter space.** Temporal β-diversity β_t was computed as the mean BC dissimilarity between time points in a time series of 1000 unit times, observed in metacommunities of $N = 64$ patches. Correlation length ϕ was varied in the range 1 to 100, environmental variability σ^2 and dispersal length ℓ in the range 10^{-2} to 1, with each parameter combination replicated 10 times. The values of ϕ, σ^2 and ℓ were each plotted on logarithmic axes. In **a** we fixed ℓ at 0.5; in **b** ϕ at 10; and in **c** σ^2 at 1.0. See Supplementary discussion for details.

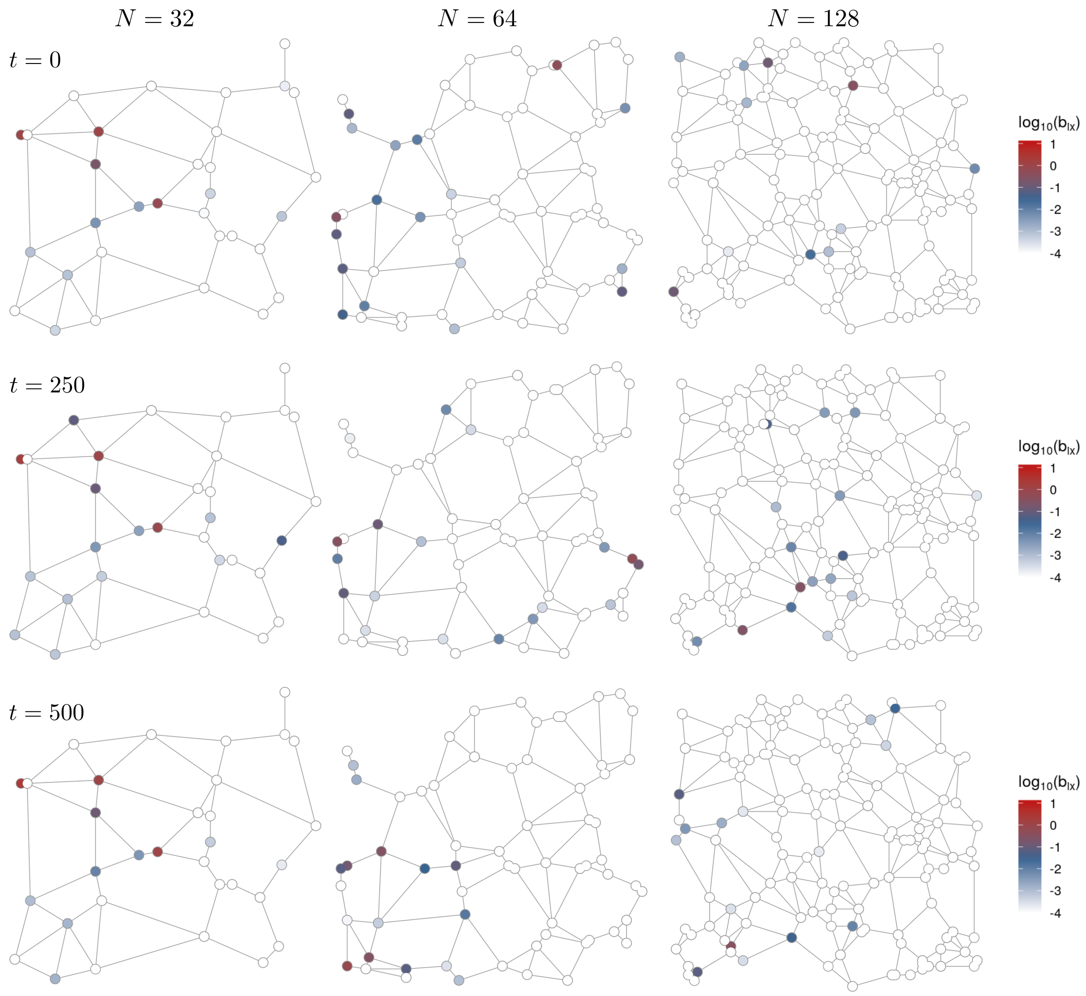

Supplementary Figure 5. **Autonomous metapopulation dynamics in large metacommunity models.** In species rich metacommunities of $N > 8$ patches, local biomasses autonomously fluctuate and the variability of those fluctuations increases with metacommunity size. Here we show the instantaneous biomass distributions for a single species in metacommunities of $N = 32$, 64 and 128, at three time points in logarithmic biomass units. For $N = 32$, autonomous fluctuations are largely restricted to the outer extremes of the species' distribution, while the core range (left of network) remains largely static. For $N = 64$, some patches or regions may be permanently occupied by the focal species, however even in this core range biomass can fluctuate by orders of magnitude. With the emergence of Gleasonian turnover in the high N limit no or few patches are permanently occupied and local community composition is no longer well characterised by the core-transient distinction[17? ?], which decomposes local communities into populations that are present almost all the time, and those observed only rarely. Hence, for $N = 128$ no obvious core range exists. Note that spatial networks are not shown to scale, the area of the model landscape is $\approx N$ in all cases. $A_{ij} = 0.5$ with probability 0.5, $\phi = 10$, $\sigma^2 = 0.01$, $\ell = 0.5$. See Main Text for details.

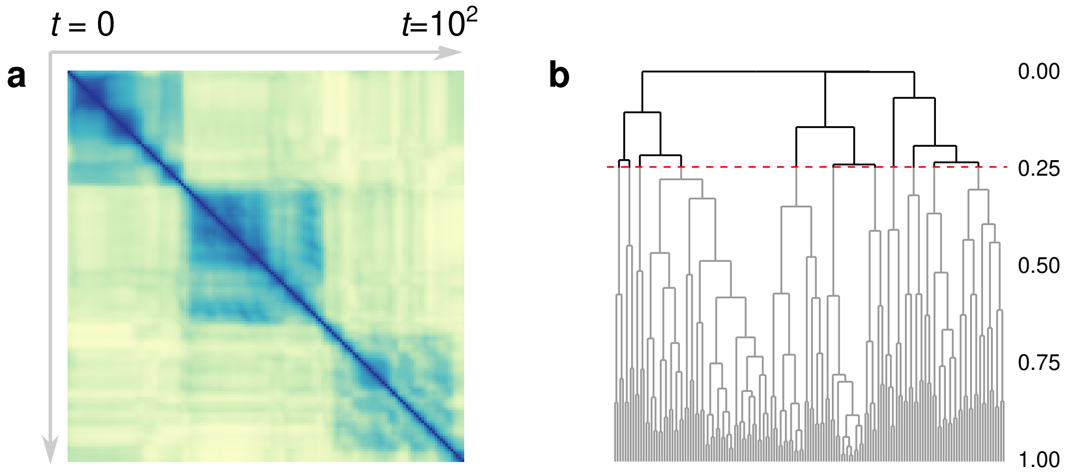

Supplementary Figure 6. **The number of compositional clusters in a community time-series analysed using hierarchical clustering.** **a**: Temporal clustering in local community composition represented by the block structure of the BC dissimilarity matrix ($N = 64$, 200 unit times shown). **b**: Using hierarchical cluster analysis we approximately quantified the number of clusters in community state using a dissimilarity threshold of 25% (red dashed line).

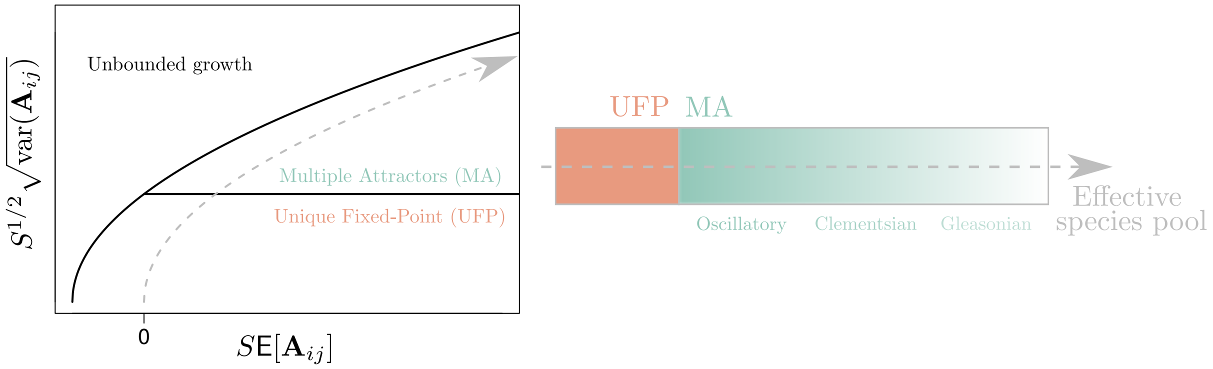

Supplementary Figure 7. **The sharp transition between UFP and MA phases.** Reproduction of the phase diagram derived by Bunin[2] showing the emergence of MA as the size S of the species pool increases. In our case, the first and second moments of the distribution in A_{ij} were fixed. Community state in phase space therefore follows a square root function with increasing S, as indicated by the dashed line. (The "Unbounded growth" phase is hence not relevant for our study.) In spatially explicit metacommunity models we observe the emergence of autonomous turnover which transitions from oscillations to Clementsian and finally Gleasonian turnover. See Supplementary discussion for details.

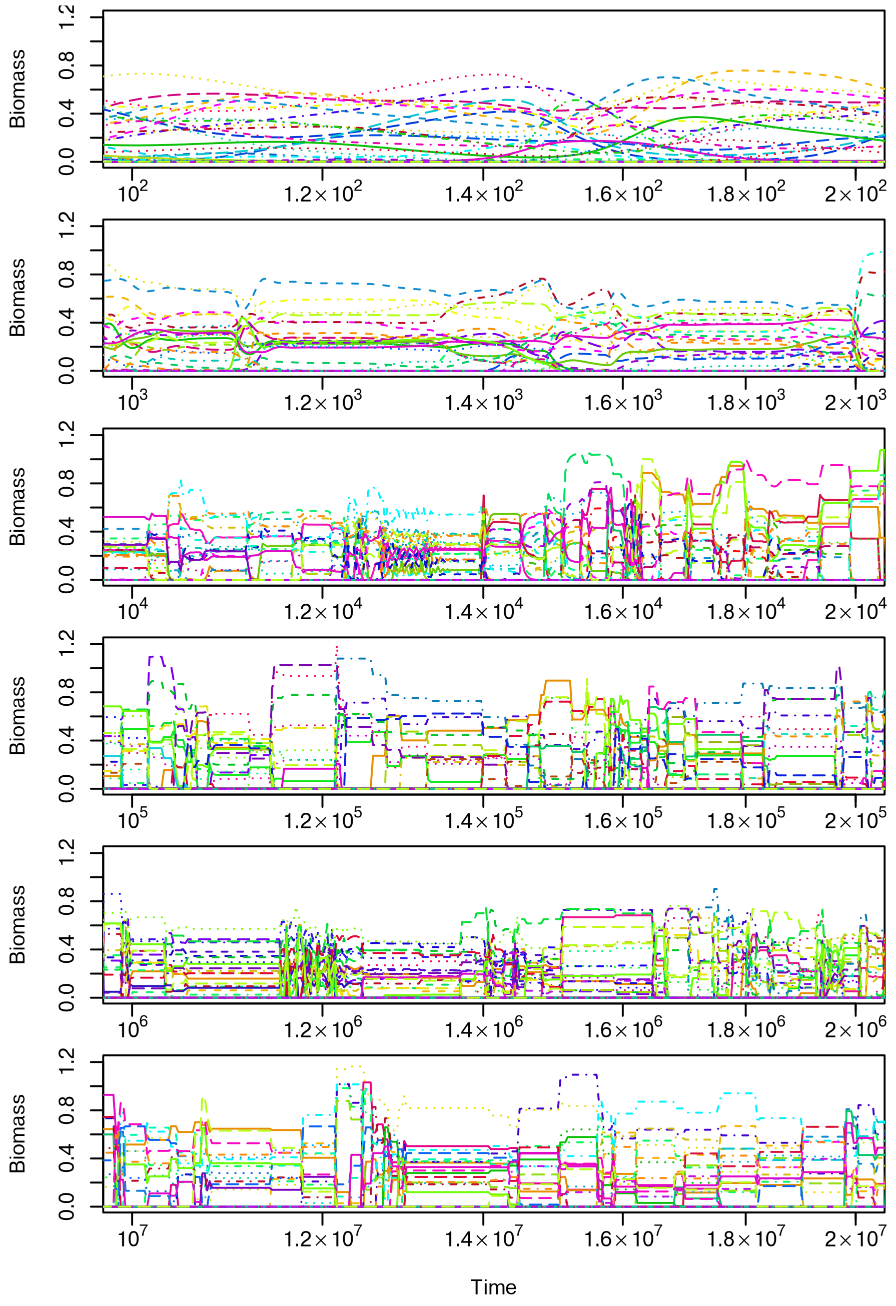

Supplementary Figure 8. **Episodes in the approach of an isolated LV community model to a heteroclinic network**. The biomasses of different species are represented by lines of different colours and style. At any moment in time, all but a few of the $S = 300$ species in the system have biomasses close to zero. With increasing simulation times t the intervals between the switches in system state, corresponding to transitions from the vicinity of one unstable equilibrium to the next, become longer, while the duration of these transitions remains of the order of magnitude of 10 time units, leading to increasingly sharper transitions on the logarithmic time scale. See Supplementary discussion for details.

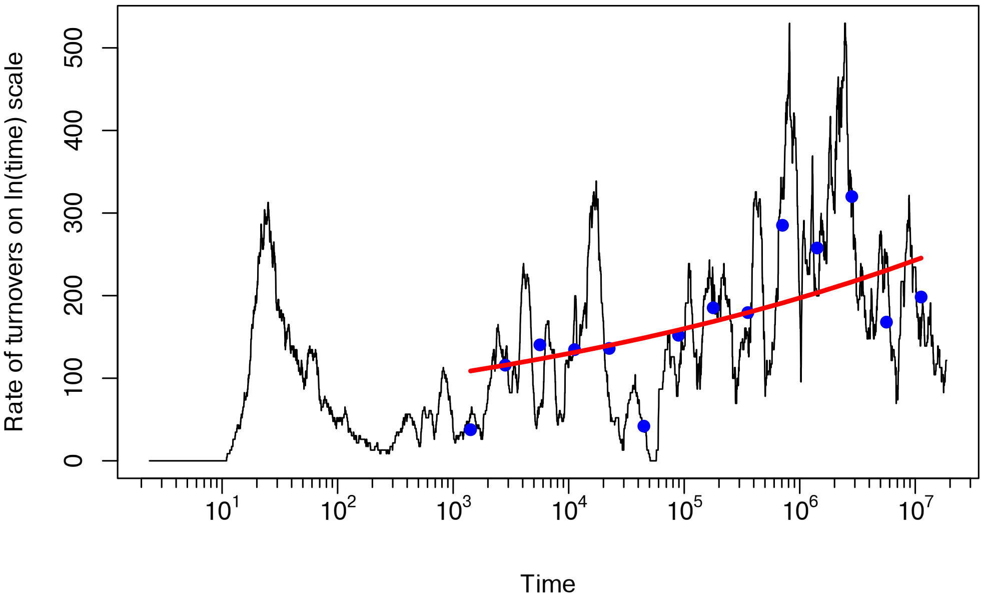

Supplementary Figure 9. **Rate of change in community composition for the simulation shown in Fig. 8**. The black line is the moving average over 100 subsequent recordings, blue dots represent averages over non-overlapping adjacent blocks of 300 recordings for $t \geq 1000$, and the red line a median nonlinear regression of the dots by a power-law (rate) $\sim t^{\nu}$ ($\nu = 0.091 \pm 0.062$, not significantly different from zero). See Supplementary discussion for details.

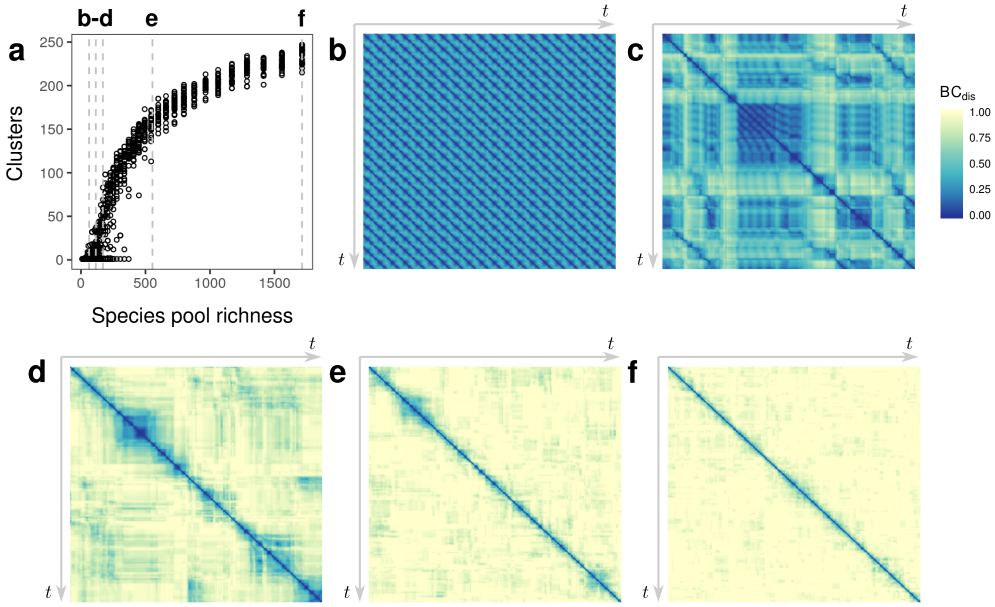

Supplementary Figure 10. **Autonomous turnover in isolated LV communities**. **a**: The number of compositional clusters detected as a function of the size of the pool of potential invaders for a propagule pressure, ϵ, of 10^{-15} biomass units per unit time. **b**-**f**: Heatmaps of the pairwise Bray-Curtis dissimilarity for the corresponding time-series (over 10^4 unit times) showing a clear transition from oscillatory to Clementsian turnover and finally to Gleasonian turnover. Dashed lines in **a** show the size of the species pool for which each community time series was generated. $A_{ij} = 0.5$ with probability 0.5, $\sigma^2 = 0.01$. The parameters ϕ and ℓ are not defined for the isolated LV models. See Supplementary discussion for details.

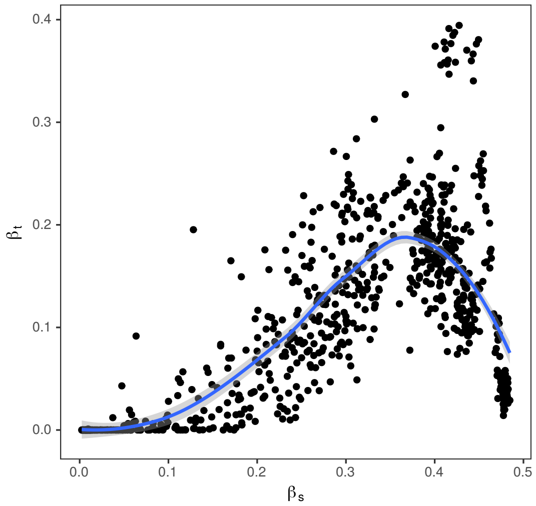

Supplementary Figure 11. **Unimodal relationship between spatial and temporal turnover.** Temporal beta diversity, computed during 1000 unit times, plotted against the spatial beta diversity of the local neighbourhood. The number of patches in a local neighbourhood depends on the patch degree, which varies. We therefore use a beta-diversity metric[6] (based on BC dissimilarity) that is normalises by the number of sites/time-points included in the sub-sample. Both β_t and β_s are averages over the metacommunity. The blue line and shaded area represent a locally weighted regression (LOESS smoothing) and 95% C.I.. Parameters N, ϕ, σ^2 and ℓ as in Fig. 4. See Supplementary discussion for details.

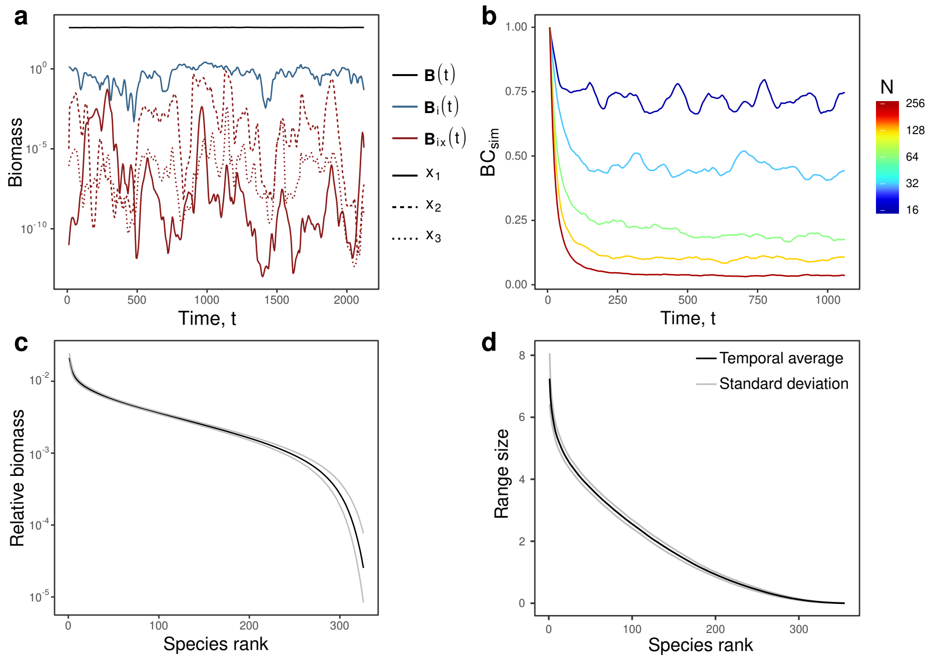

Supplementary Figure 12. **Temporally robust community structure a**: We highlight the scale dependence of autonomous population dynamics by showing the biomass of three random local populations of the same species (B_{ix}, red), of the metapopulation of which they form a part ($\mathrm{B}_i = \sum_x \mathbf{B}_{ix}$, blue) and finally of the entire metacommunity ($\mathrm{B} = \sum_i \sum_x \mathbf{B}_{ix}$), black). **b**: Autonomous turnover can be substantial. Here we show the decay of spatially averaged BC *similarity* from an arbitrary initial composition in metacommunities of $N = 16$, 32, 64, 128, and 256 patches. For large metacommunities undergoing autonomous Gleasonian turnover, the percentage of permanent populations, and hence the temporal BC similarity can drop to zero. **c**: Metacommunity scale relative rank abundance curve, plotted with species 'identity' disregarded. The black curve represents the mean biomass observed at a given rank, while grey curves represent the mean $\pm$ one standard deviation. This figure highlights the temporally invariant diversity structure at the metacommunity scale. **d**: The temporally averaged rank range size curve, plotted as in **c**. $A_{ij} = 0.5$ with probability 0.5, $\phi = 10$, $\sigma^2 = 0.01$, $\ell = 0.5$. $N = 64$ for **a**, **c** and **d**. See Supplementary discussion for details.

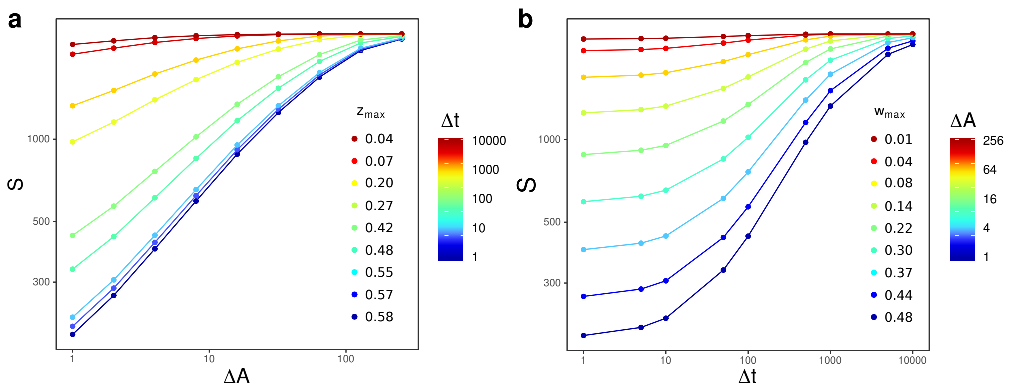

Supplementary Figure 13. **The Species-Time-Area-Relation.** The nested SAR (**a**) and STR (**b**) generated using a sliding window approach for a single metacommunity model of $N = 256$. Metacommunity models are closed systems and as such, both the SAR and STR saturate for the large sub-samples. Therefore, we defined the exponents of the STAR by the maximum slopes observed on double logarithmic axes. $A_{ij} = 0.5$ with probability 0.5, $\phi = 10$, $\sigma^2 = 0.01$, $\ell = 0.5$. See Supplementary discussion for details.

[1] Rossberg, A. G. *Food Webs and Biodiversity: Foundations, Models, Data* (John Wiley & Sons, 2013).

[2] Bunin, G. Ecological communities with Lotka-Volterra dynamics. *Physical Review E* **95** (2017).

[3] Barbier, M., Arnoldi, J.-F., Bunin, G. & Loreau, M. Generic assembly patterns in complex ecological communities. *Proceedings of the National Academy of Sciences* **115**, 2156–2161 (2018).

[4] O'Sullivan, J., Terry, C. & Rossberg, A. G. Intrinsic ecological dynamics drive biodiversity turnover in model metacommunities: Supporting data `https://doi.org/10.6084/m9.figshare.14139644.v1` (2021).

[5] O'Sullivan, J. D., Knell, R. J. & Rossberg, A. G. Metacommunity-scale biodiversity regulation and the self-organised emergence of macroecological patterns. *Ecology Letters* **22**, 1428–1438 (2019).

[6] Legendre, P. & De Cáceres, M. Beta diversity as the variance of community data: dissimilarity coefficients and partitioning. *Ecology letters* **16**, 951–963 (2013).

[7] White, E. P. *et al.* A comparison of the species-time relationship across ecosystems and taxonomic groups. *Oikos* **112**, 185–195 (2006).

[8] Shurin, J. B. *et al.* Diversity–stability relationship varies with latitude in zooplankton. *Ecology Letters* **10**, 127–134 (2007).

[9] Ptacnik, R. *et al.* Diversity predicts stability and resource use efficiency in natural phytoplankton communities. *Proceedings of the National Academy of Sciences* **105**, 5134–5138 (2008).

[10] Ptacnik, R., Andersen, T., Brettum, P., Lepistö, L. & Willén, E. Regional species pools control community saturation in lake phytoplankton. *Proceedings of the Royal Society B: Biological Sciences* **277**, 3755–3764 (2010).

[11] Korhonen, J. J., Soininen, J. & Hillebrand, H. A quantitative analysis of temporal turnover in aquatic species assemblages across ecosystems. *Ecology* **91**, 508–517 (2010).

[12] Stegen, J. C. *et al.* Stochastic and deterministic drivers of spatial and temporal turnover in breeding bird communities. *Global Ecology and Biogeography* **22**, 202–212 (2013).

[13] Hofbauer, J. Heteroclinic cycles in ecological differential equations. *Equadiff 8* 105–116 (1994).

[14] Doak, D. F. *et al.* The statistical inevitability of stability-diversity relationships in community ecology. *The American Naturalist* **151**, 264–276 (1998).

[15] Tilman, D., Lehman, C. L. & Bristow, C. E. Diversity-stability relationships: statistical inevitability or ecological consequence? *The American Naturalist* **151**, 277–282 (1998).

[16] Yachi, S. & Loreau, M. Biodiversity and ecosystem productivity in a fluctuating environment: the insurance hypothesis. *Proceedings of the National Academy of Sciences* **96**, 1463–1468 (1999).

[17] Magurran, A. E. & Henderson, P. A. Temporal turnover and the maintenance of diversity in ecological assemblages. *Philosophical Transactions of the Royal Society B: Biological Sciences* **365**, 3611–3620 (2010).
