# Supplementary material for: Intrinsic ecological dynamics drive biodiversity turnover in model metacommunities
Source: Nat Commun. 2021 Jun 15;12:3627. doi: 10.1038/s41467-021-23769-7 (PMC8206366; doi:10.1038/s41467-021-23769-7)
Supplement: Supplementary file 2 — Reporting Summary [file 41467_2021_23769_MOESM2_ESM.pdf]

nature research

Corresponding author(s): Jacob Dinner O'Sullivan

Last updated by author(s): 12-4-2021

Reporting Summary

Nature Research wishes to improve the reproducibility of the work that we publish. This form provides structure for consistency and transparency in reporting. For further information on Nature Research policies, see our Editorial Policies and the Editorial Policy Checklist.

Statistics

For all statistical analyses, confirm that the following items are present in the figure legend, table legend, main text, or Methods section.

n/a	Confirmed	
☐	☒	The exact sample size (n) for each experimental group/condition, given as a discrete number and unit of measurement
☐	☒	A statement on whether measurements were taken from distinct samples or whether the same sample was measured repeatedly
☒	☐	The statistical test(s) used AND whether they are one- or two-sided<br>*Only common tests should be described solely by name; describe more complex techniques in the Methods section.*
☐	☒	A description of all covariates tested
☒	☐	A description of any assumptions or corrections, such as tests of normality and adjustment for multiple comparisons
☐	☒	A full description of the statistical parameters including central tendency (e.g. means) or other basic estimates (e.g. regression coefficient) AND variation (e.g. standard deviation) or associated estimates of uncertainty (e.g. confidence intervals)
☒	☐	For null hypothesis testing, the test statistic (e.g. F, t, r) with confidence intervals, effect sizes, degrees of freedom and P value noted<br>*Give P values as exact values whenever suitable.*
☒	☐	For Bayesian analysis, information on the choice of priors and Markov chain Monte Carlo settings
☒	☐	For hierarchical and complex designs, identification of the appropriate level for tests and full reporting of outcomes
☒	☐	Estimates of effect sizes (e.g. Cohen's d, Pearson's r), indicating how they were calculated

Our web collection on statistics for biologists contains articles on many of the points above.

Software and code

Policy information about availability of computer code

Data collection	Data collection was done using a purpose built software package publicly available here: https://github.com/jacobosullivan/LVMCM_src.<br>The software links to pre-built libraries including SUNDIALS, Armadillo and Boost
Data analysis	Data were analysed using the R environment for statistical computing and visualisation (version 4.0.3)

For manuscripts utilizing custom algorithms or software that are central to the research but not yet described in published literature, software must be made available to editors and reviewers. We strongly encourage code deposition in a community repository (e.g. GitHub). See the Nature Research guidelines for submitting code & software for further information.

Data

Policy information about availability of data

All manuscripts must include a data availability statement. This statement should provide the following information, where applicable:
- Accession codes, unique identifiers, or web links for publicly available datasets
- A list of figures that have associated raw data
- A description of any restrictions on data availability

Simulation data supporting a subset of the results of this study are available at https://doi.org/10.6084/m9.figshare.14139644.v1

Field-specific reporting

Ecological, evolutionary & environmental sciences study design

All studies must disclose on these points even when the disclosure is negative.

Study description	In this study we explore the phenomenology and mechanisms involved in autonomous temporal change in model metacommunities.
Research sample	We generated simulated metacommunities of random species, varying several key parameters. The models are not designed to represent any specific ecosystem.
Sampling strategy	We chose ten-fold replication of each combination of parameters and a large number of combinations (around 600). Being a deterministic model in terms of the ecological dynamics included, variance for a given parameter combination is typically low. In several figures we show data for each site in each model metacommunity which verifies this.
Data collection	As a modelling study, all data collection was done was done computationally using a bespoke simulation (code available). We also use publicly available data from published sources for comparison.
Timing and spatial scale	The effect of spatial scale is explicitly explored by varying the number of sites and the total landscape area. We take steps to ensure that time-scale does not impact interpretation. These involve long relaxation times to avoid extinction debts and fixing time series length for all simulations.
Data exclusions	No data were excluded
Reproducibility	The software used is publicly available and therefore the study could be readily reproduced. In the Github repo we include explicit instruction on how to reproduce a key subset of the results which demonstrates the emergence of autonomous turnover during metacommunity assembly and confirms our analytic prediction that autonomous turnover occurs precisely when local species richness limits are reached.
Randomization	Results were grouped according to parameterisation. Within each group, landscape topography, environmental responses and biotic interaction coefficients were all randomly sampled.
Blinding	As a theoretical study, blinding was not used.

Did the study involve field work? ☐ Yes ☒ No

Reporting for specific materials, systems and methods

We require information from authors about some types of materials, experimental systems and methods used in many studies. Here, indicate whether each material, system or method listed is relevant to your study. If you are not sure if a list item applies to your research, read the appropriate section before selecting a response.

Materials & experimental systems

n/a	Involved in the study
☒	☐ Antibodies
☒	☐ Eukaryotic cell lines
☒	☐ Palaeontology and archaeology
☒	☐ Animals and other organisms
☒	☐ Human research participants
☒	☐ Clinical data
☒	☐ Dual use research of concern

Methods

n/a	Involved in the study
☒	☐ ChIP-seq
☒	☐ Flow cytometry
☒	☐ MRI-based neuroimaging
